# Legal Notes for Pharmacists

**Published:** 1915-11-13

**Authors:** 


### LEGAL NOTES FOR PHARMACISTS.

One of the contributions to the current number of the *Journal of the Society of Comparative Legislation* is an interesting comparative study of British and foreign Pharmacopœias. The subject is one on the legal aspects of which light is rarely cast, and we take the opportunity of endeavouring to focus its position in law, tendering our respectful acknowledgments to the author of the article in question (Mr. H. Wippell Gadd) for suggesting the subject and setting out fully the facts and opinions, which we now briefly summarise.

By the Medical Act of 1858 the duty was cast upon the General Medical Council of publishing a book, to be called the British Pharmacopœia, but this Act did not make the book binding on anybody. By the Medical Act of 1862 exclusive rights were given to the General Medical Council of publishing, printing, and selling the British Pharmacopœia, and any Act of Parliament, Order in Council, or custom relating up to that time to the London, Edinburgh, and Dublin Pharmacopœias was to be deemed to refer to the Pharmacopœia published by the General Medical Council.

The General Medical Council has no disciplinary powers over pharmacists or dispensers. Legal position and authority are, however, given to the British Pharmacopœia by Section 15 of the Pharmacy Act of 1868, by which a penalty is provided against any person who compounds any medicines of the British Pharmacopœia except according to the formularies of the said Pharmacopœia. It has been found impossible to control compounding, and the above provision is a dead letter; curiously enough, the enforcement of the standards of the Pharmacopœia has been effected, so far as it has been effected at all, by means of an Act in which there is no special reference to it whatever, the Sale of Food and Drugs Act, the general scheme of which is to penalise the seller of an article which is not of the nature, quality, and substance demanded by the purchaser. Under that Act there have been several leading cases of which the general effect is that when a compounded medicine is demanded by a name which occurs in the Pharmacopœia, an article made in accordance with the formula, and answering the tests, if any, of the Pharmacopœia, is presumed to be required, and only very strong evidence to the contrary will suffice to rebut such a presumption.

These cases, however, refer to simple commercial transactions—that is to say, the simple sale of medicinal preparations, a very different matter from the dispensing of physicians' prescriptions. In the latter case the duty in law of the dispenser is thought to be this, that he translate the prescription and make up the medicine in accordance with what appears to him in the light of his technical training and experience to be the intention of the prescriber. But, and we here quote from our authority, "the intention of the prescriber is not always clear, for the reason that medicines with names well known to the medical profession may and do have their composition and strength materially altered in successive editions of the Pharmacopœia, and each issue of the British Pharmacopœia becomes authoritative coincidentally with its publication. A busy professional man, such as the average general medical practitioner, cannot always be expected at once to be cognisant of and still less to have assimilated sudden changes in the tools with which he has worked during perhaps the whole of his active career, and it follows, therefore, that a prescriber may be thinking of an obsolete strength and dose when specifying a certain medicinal preparation and may order it in inappropriate and even dangerous quantities. The dispenser is not, therefore, necessarily or always bound to make up a prescription with ingredients compounded in accordance with the formulæ and answering the tests and conforming to the standards of the current Pharmacopœia." The suggestion is made that to mitigate the possible danger above referred to an interval of, say, six months might be allowed to elapse after publication of each new edition of the Pharmacopœia before it came into force.

### Pharmacopœia Revision.

The remaining principal point referred to in the article under notice is the curious fact that, though the compounding and dispensing of medicine has been practically given up by medical men, the sole responsibility for the production of the Pharmacopœia ultimately rests in this country with a Committee appointed by the General Medical Council. It is true that there is a Committee of Reference in Pharmacy appointed by the Pharmaceutical Society, but final decisions in every case rest with the Pharmacopœia Committee of the General Medical Council. At the International Congress of Pharmacy in Brussels it was stated that Great Britain appeared to be the only country in which the National Pharmacopœia was produced by a Commission from which pharmacists were excluded.

The foregoing, we think, indicates pretty well the legal aspects of the British Pharmacopœia. It is urged that fresh legislation for its reform is required, and that this should be prepared by an authoritative Commission consisting of medical men, pharmacists, and analysts drawn from and representative of the whole Empire. Our task, however, is to state the law as it is, of the necessity of the proposed reform our readers are the better judges.

